# Characteristics and risk factors of type I or II endoleaks after thoracic endovascular aortic repair and open surgery

**DOI:** 10.3389/fcvm.2025.1489023

**Published:** 2025-02-28

**Authors:** Fan Zhu, Jia Chen, Yuanyuan Guo, Chang Shu

**Affiliations:** ^1^ Department of Vascular Surgery, Fuwai Yunnan Cardiovascular Disease Hospital, Affiliated Cardiovascular Hospital of Kunming Medical University, Kunming, China; ^2^ Department of Vascular Surgery, The Second Xiangya Hospital of Central South University, Changsha, China; ^3^ Vascular Diseases Institute of Central South University, The Second Xiangya Hospital of Central South University, Changsha, China; ^4^ Department of Laboratory Medicine, Longhua Hospital, Shanghai University of Traditional Chinese Medicine, Shanghai, China

**Keywords:** thoracic endovascular aortic repair, type I endoleak, type II endoleak, left subclavian artery, risk factors

## Abstract

**Background:**

Type I endoleaks (T1ELs) and type II endoleaks (T2ELs) are among the most severe complications that occur after thoracic endovascular aortic repair (TEVAR) and open surgery. This investigation aimed to analyze the predictors and multiple risk factors of T1ELs and T2ELs, with a particular focus on the diameter of the false lumen and the pathology of the left subclavian artery (LSA).

**Methods:**

A total of 245 patients (mean age 57 ± 13 years) who had undergone open surgery or TEVAR were recruited and followed for a mean of 18 ± 10 months. Seven patients (2.8%) were classified into the T1EL group, and another seven (2.8%) were classified into the T2EL group. Contrast-enhanced computed tomography angiography of the entire aorta confirmed the diagnosis of aortic disease (e.g., thoracic aortic dissection, thoracic aortic aneurysm, and/or type B intramural hematoma) as well as the presence of T1ELs or T2ELs.

**Results:**

Reoperation was more common in the T1EL group (*n* = 4; 57%) than in the T2EL group (*n* = 2; 29%); also, reintervention for stenting of the thoracic endovascular graft was more frequent in the T1EL group (4 vs. 1). In T1EL group, four patients (57%) accepted reoperation due to progressive enlargement of the false lumen’s diameter (aneurysm size > 55 mm) (*n* = 4; 100%) and sharp, persistent chest and back pain (*n* = 4; 100%). In the T2EL group, two patients (29%) required reintervention due to the false lumen’s growth rate (>5 mm in diameter per half year) and symptoms of pulmonary vascular compression such as hemoptysis and dyspnea (*n* = 2; 100%). The median survival rate of patients in the T1EL and T2EL groups was 31 months [95% confidence interval (CI) 0.0937–11.4] and 30 months (95% CI: 0.08775–10.67), respectively. The proximal opening angle of the LSA (OR 3.141, 95% CI: 2.615–3.773) was significantly associated with the incidence of T1EL. Both the proximal opening angle of the LSA and its diameter were significantly associated with the occurrence of T2ELs.

**Conclusions:**

To prevent the occurrence of T1ELs and T2ELs, appropriate stent grafts and the pathology of the LSA should be carefully considered.

## 1 Introduction

Thoracic endovascular aortic repair (TEVAR) has become the gold standard for the treatment of thoracic aortic dissection (TAD) (1). However, its long-term stability remains unknown. Its complications may include retrograde type A aortic dissection, stroke, paraplegia, endoleaks, and even death (2, 3). Endoleaks are categorized based on their anatomical location and underlying causes and divided into four distinct types in TEVAR. A type I endoleak (T1EL) occurs when the endograft fails to adequately adhere to the vessel wall at the attachment site. It is further categorized into three subtypes: (Ia) for proximal endoleaks, (Ib) for distal attachment site endoleaks, and (Ic) for insufficient sealing by an iliac occlude plug in aorto-uni-iliac repairs with a crossover graft. A type II endoleak (T2EL) is characterized by the perfusion of the aneurysm sac from collateral vessels. In recent decades, endoleaks have been recognized as the most common complication of TEVAR, with their incidence rate ranging from 23.3% to 32.9% (4, 5). The 2022 guidelines of the American Association for Thoracic Surgery clinical practice indicate that after a TEVAR procedure, the overall rate of T1ELs is 16%, while T2ELs occur in 4% of cases (1). Many reports also indicate that T1ELs can lead to a persistent flow through the false lumen (FL), retrograde dissection, and aortic rupture (6–9). In addition, T2ELs can lead to late aneurysmal dilation and rupture (6–9).

T1ELs and T2ELs remain challenging for surgeons, as both are difficult to prevent and cure. Open surgery [e.g., hypothermic cardiopulmonary bypass (CPB), stented elephant trunk] and/or reinterventional surgery (e.g., repeated TEVAR, branched stent graft) would be required to manage these complications. Embolization of the false lumen has been used in treating both T1ELs and T2ELs. However, the long-term follow-up results from many reports have been controversial (10, 11). Hence, the unsatisfactory results of previous strategies have suggested a new approach: addressing relevant risk factors preoperatively and tackling them afterward. The purpose of the present study was to analyze the potential risks of T1ELs and T2ELs, with a particular focus on the pathologic arch branches, including the anatomy of the left subclavian artery (LSA).

## 2 Materials and methods

### 2.1 Patients

From November 2018 to March 2022, we reviewed 245 patients who underwent TEVAR/open surgery (type II hybrid arch repair) at our department. This was a single-center study. Data from 14 patients with type I or II endoleaks were retrospectively collected and analyzed. Among them, 11 patients were referred to our center between 1 week and 5 years after undergoing TEVAR at another hospital, whereas 3 patients were evaluated 1 week after undergoing TEVAR at our center. The study was conducted in accordance with the Declaration of Helsinki, and the protocol was approved by the Institutional Ethics Review Board of Fuwai Yunnan Cardiovascular Disease Hospital (approval number: 2022-071-01) without the need for patient informed consent. All patients were enrolled in a follow-up program that included periodic visits by a dedicated team at 1, 6, and 12 months after surgery, followed by annual assessments. Computed tomography angiography (CTA) was used to confirm the diagnosis of aortic disease [e.g., TAD, thoracic aortic aneurysm (TAA), and/or type B intramural hematoma (IMH)] and the presence of T1ELs or T2ELs. In this study, a T2EL was defined as persistent retrograde FL perfusion from the intercostal artery or the LSA. Patients undergoing open surgery/TEVAR for type A intramural hematoma, traumatic aortic transection, syphilitic aortic aneurysm, and tuberculosis aortic pseudoaneurysm were excluded from this retrospective study (7, 8). Another inclusion criterion comprised the availability of preoperative and postoperative CTA images with slice thicknesses of 6–8 mm. Vascular pathologies included TAD in 154 patients, TAA in 35 patients, and type B IMH in 56 patients (Table 1). The follow-up period was 18 ± 10 months. All patients were diagnosed and categorized into two groups on the basis of CTA. There were seven patients (2.8%) in the T1EL group and seven patients (2.8%) in the T2EL group. The mean age of the T1EL group was 65 ± 14 years; all patients were men; in this group, four patients agreed to reoperation and repair, and one patient died. The mean age of the T2EL group was 64 ± 10 years; 71% were men, while 29% were women; in this group, two patients agreed to reoperation and repair, and one patient died.

**Table 1:** Baseline characteristics of 245 patients.

Variable	Total (*n* = 245)	Endoleak (*n* = 14)		*P*-value
		T1EL group (*n* = 7; 2.8%)	T2EL group (*n* = 7; 2.8%)	
Mean age (years)	57 ± 13	65 ± 14	64 ± 10	0.572
Male (%)	193 (79%)	7 (100%)	5 (71%)	0.127
BMI	25 ± 4.6	24 ± 3	25 ± 3	0.292
Follow-up period (months)	18 ± 10	17 ± 11	13 ± 9	None
Hypertension (%)	233 (95%)	7 (100%)	6 (86%)	0.127
Diabetes (%)	19 (7.8%)	1 (14%)	0	0.299
CAD (%)	60 (24.5%)	7 (100%)	4 (57%)	0.051
Cerebrovascular disease (%)	20 (8%)	0	0	None
History of smoking^a^ (%)	155 (63%)	7 (100%)	4 (57%)	0.051
Aortic pathology				
TAD	154 (63%)	6 (86%)	5 (71%)	0.515
TAA	35 (14%)	1 (14%)	2 (29%)	0.515
IMH (type B)	56 (23%)	—	—	None

BMI, body mass index; CAD, coronary artery disease; IMH, intramural hematoma; T1EL, type I endoleak; T2EL, type II endoleak; TAA, thoracic aortic aneurysm; TAD, thoracic aortic dissection.

^a^ Includes current and former smokers.

### 2.2 Modality and measurements

All patients underwent contrast-enhanced CTA of the whole aorta with 3D reconstruction; an image-processing workstation (Siemens CT SOMATOM Definition, Germany) was used to diagnose the disease by the same evaluator, classify the type of endoleak, evaluate the proximal opening angle and diameter of the LSA, measure the distance between the midline of the coronary plane of the aorta aortic and the midline of the LSA sagittal plane, and analyze the aortic aneurysm. The angle was measured between the sagittal plane of the LSA and the aortic arch in the coronal section. Multidetector CT (MDCT) images with a slice thickness of 6–8 mm were acquired.

### 2.3 Surgical technique

There were four different surgical approaches for treating T1ELs and T2ELs after TEVAR/open surgery, including group (1) TEVAR, (2) TEVAR with revascularization of the LSA and fenestration *in vitro*, (3) TEVAR with revascularization of the left common carotid artery (LCCA) using a single-chimney stent, and (4) type II hybrid arch repair with a coronary artery bypass graft and LSA embolization. The procedures for groups (1)–(3) were strictly based on the method described by Shu et al. (12). All patients who received TEVAR were carefully selected on the basis of a suitable proximal landing zone (LZ). In most cases, the LZ needed to be more than 15 mm from the target branches of the aortic arch and have a diameter between 20 and 45 mm. If these criteria were not met, we selected a type II hybrid arch repair (13).

### 2.4 Type II hybrid aortic arch repair

This operation was performed using CPB, with the lowest rectal temperature set at 28 °C. A four-branched vascular prosthesis (Maquet Cardiovascular, Wayne, NJ) was placed after transecting the ascending aorta. Then, the distal end of the vascular prosthesis was anastomosed to the stump of the aortic arch stump, and the innominate artery, LCCA, and LSA were anastomosed to the branches of the prosthesis. Next, we deployed the endograft in zone 0. The stent graft was oversized by 10% (14–16).

### 2.5 Statistical analysis

Continuous variables were presented as means ± standard deviations or medians (minimum–maximum range) and compared using Welch's *t*-test. Categorical variables were presented as frequencies with percentages and compared using Fisher's test. Overall survival curves for T1EL and T2EL were estimated using the Kaplan–Meier method and compared with the log-rank test. Univariable and multivariable logistic regression analyses were performed to examine the risk factors for a bird-beak configuration on the first postoperative MDCT. All statistical analyses were conducted using IBM SPSS Statistics (version 19.0). Differences with a *P-*value <0.05 were considered statistically significant. The level of significance was set at 5%. Quantitative data were presented as percentages or means (±standard errors). The chi-square or Welch's unpaired *t*-test was used to compare categorical variables. Results were expressed as odds ratios (ORs) and corresponding 95% confidence intervals (CIs) (17).

## 3 Results

### 3.1 Patient profile

From November 2018 to March 2022, a total of 245 patients with TAA, TAD, and/or type B IMH were enrolled for TEVAR. The patients had a mean age of 57 ± 13 years, with 193 (79%) being men and 52 (21%) being women. Their mean BMI was 25 ± 4.6. Their pathologies were as follows: 154 (63%) had TAD, 35 (14%) had TAA, and 56 (23%) had type B IMH. Among the total patients, seven (2.8%) had T1ELs and seven (2.8%) had T2ELs. There was no significant difference between the two groups in terms of mean age, male population, BMI, follow-up period, hypertension, diabetes, coronary artery disease, cerebrovascular disease, history of smoking, or aortic pathology (Table 1). Other characteristics of patients with T1ELs and T2ELs are summarized in Tables 2, 3.

**Table 2:** Clinical profiles of endoleak patients.

No.	Sex	Age	Endoleak	Involved vessels	Aortic type	Clinical symptoms	Stent graft and first operation	Stent graft and second operation	Follow-up result
1	M	55	Ib	—	I	—	Cook Zenith TX2, 34-200; TEVAR	Medtronic Valiant 36-200, 34-200; TEVAR	Survived
2	M	48	Ib	Intercostal artery	III	Impending rupture	Medtronic Valiant, 28-200; TEVAR	Lifetech Ankura 30-26-160; TEVAR	Survived
3	M	79	Ia	LSA	II	—	Medtronic Valiant, 30-160; TEVAR	Lifetech Ankura 40-32-200, 32-24-160, Biotronic PRO-Kinetic Energy Explorer 8-38-130; TEVAR + revascularization of the LSA with fenestration *in vitro*	Died
4	M	77	Ia	LSA	II	Impending rupture	Lifetech Ankura 30-22-200; TEAVR + revascularization of the LSA with fenestration *in vitro*	MAQUET 28-10-8-8-10, Medtronic Valiant 32-200; type II hybrid arch repair + CABG	Survived
5	M	80	Ia	LSA	III	—	MicroPort Castor, C342810-2002510; TEAVR	—	Survived
6	M	65	Ib	—	III	—	Lifetech Ankura 30-22-200, Biotronic 8-38; TEAVR + revascularization of the LSA with fenestration *in vitro*	—	Survived
7	M	49	Ia	LSA	III	—	Lifetech Ankura, 36-28-200; TEAVR	—	Survived
8	M	75	II	Intercostal artery	III	—	Medtronic Valiant 40-200, Bard fluency 6-60, 12-40, 6-60, GORE VIABAHN 9-150; TEAVR + interventional revascularization of supra-aortic branches	—	Survived
9	F	79	II	LSA	III	—	Lifetech Ankura 32-26-180, MicroPort Cuff 28-80, Biotronic PRO-Kinetic Energy Explorer 10-30; TEAVR + revascularization of the LSA with fenestration *in vitro*	—	Died
10	F	61	II	Intercostal artery	II	—	Lifetech Ankura 28-22-200, MicroPort Cuff 28-80; TEAVR	—	Survived
11	M	68	II	LSA	I	—	Lifetech Ankura 30-22-200; TEAVR	Medtronic Valiant 34-200, Bard fluency 8-60; TEAVR + revascularization of the LCCA with a single chimney	Died
12	M	57	II	Intercostal artery	II	—	Lifetech Ankura 30-22-200; TEAVR		Survived
13	M	57	II	LSA	II	—	Lifetech Ankura 34-26–200; TEAVR + transposition of the LCCA-LSA	Boston Scientific interlock8-20; LSA embolization	Survived
14	M	50	II	Intercostal artery	I	—	Lifetech Ankura 30-22-200; TEAVR	—	Survived

CABG, coronary artery bypass grafting; M, male; F, female; LCCA, left common carotid artery; LSA, left subclavian artery.

**Table 3:** Clinical profiles of endoleak patients.

No.	Proximal opening angle of LSA	Diameter of the LSA (mm)	Distance between the LSA and the aortic arch in the coronal section	Aneurysm size >55 mm
1	53	9	4	Y
2	33	10	1	Y
3	38	9	10	Y
4	46	7	7	Y
5	55	9	7	N
6	43	7	8.5	N
7	52	6	9	N
8	18	9	1	Y
9	11	8.5	3	Y
10	17	9	5	Y
11	23	8	7	Y
12	35	8	10	N
13	47	10	8	Y
14	40	8	8.5	Y

### 3.2 Surgical data

**Figure 1:**
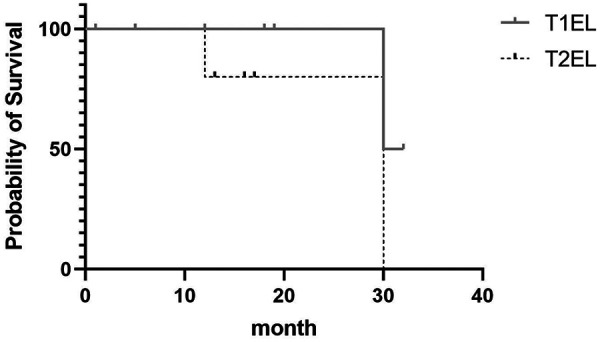
Kaplan–Meier survival curves showing the overall survival of patients with T1EL and T2EL. Their median survival rate was 31 and 30 months, respectively.

Surgical details and follow-up during the period of reoperation and/or reintervention for T1EL/T2EL patients are summarized in Tables 2, 3 and Figure 1. T1EL data indicate that four patients (57%) agreed to reoperation due to progressive enlargement of the false lumen (aneurysm >55 mm) as well as sharp, persistent chest and back pain. The surgical procedures included TEVAR in two patients (50%), TEVAR plus LSA revascularization with fenestration *in vitro* in one patient (25%), and type II hybrid arch repair plus coronary artery bypass grafting (CABG) in one patient (25%). We chose open surgery over reintervention in cases lacking an adequate LZ. Unfortunately, patient No. 3 from the T1EL group, who underwent reoperation, passed away after 30 months (Table 2).

In the T2EL group, two patients (29%) underwent reintervention due to the growth of the false lumen's diameter (>5 mm per half year) and symptoms of pulmonary vascular compression, such as hemoptysis and dyspnea. One patient underwent TEVAR and revascularization LSA with fenestration *in vitro* but died from a T2EL after 12 months. One patient underwent TEVAR plus LCCA revascularization through the single-chimney technique but also died from a T2EL after 30 months (Tables 2, 3). Another patient was successfully treated with LSA embolization. The remaining five patients are still alive and are being regularly monitored with CTA of the entire aorta.

Figure 1 shows the Kaplan–Meier curve indicating the rate of progression of T1ELs and T2ELs. In the T1EL group, the median survival time was 31 months (95% CI: 0.0937–11.4); in the T2EL group, the median survival time was 30 months (95% CI: 0.08775–10.67). The *P-*value (0.2482) indicates no significant difference.

### 3.3 Multivariate risk factors for T1ELs/T2ELs on multidetector computed tomography

**Figure 2:**
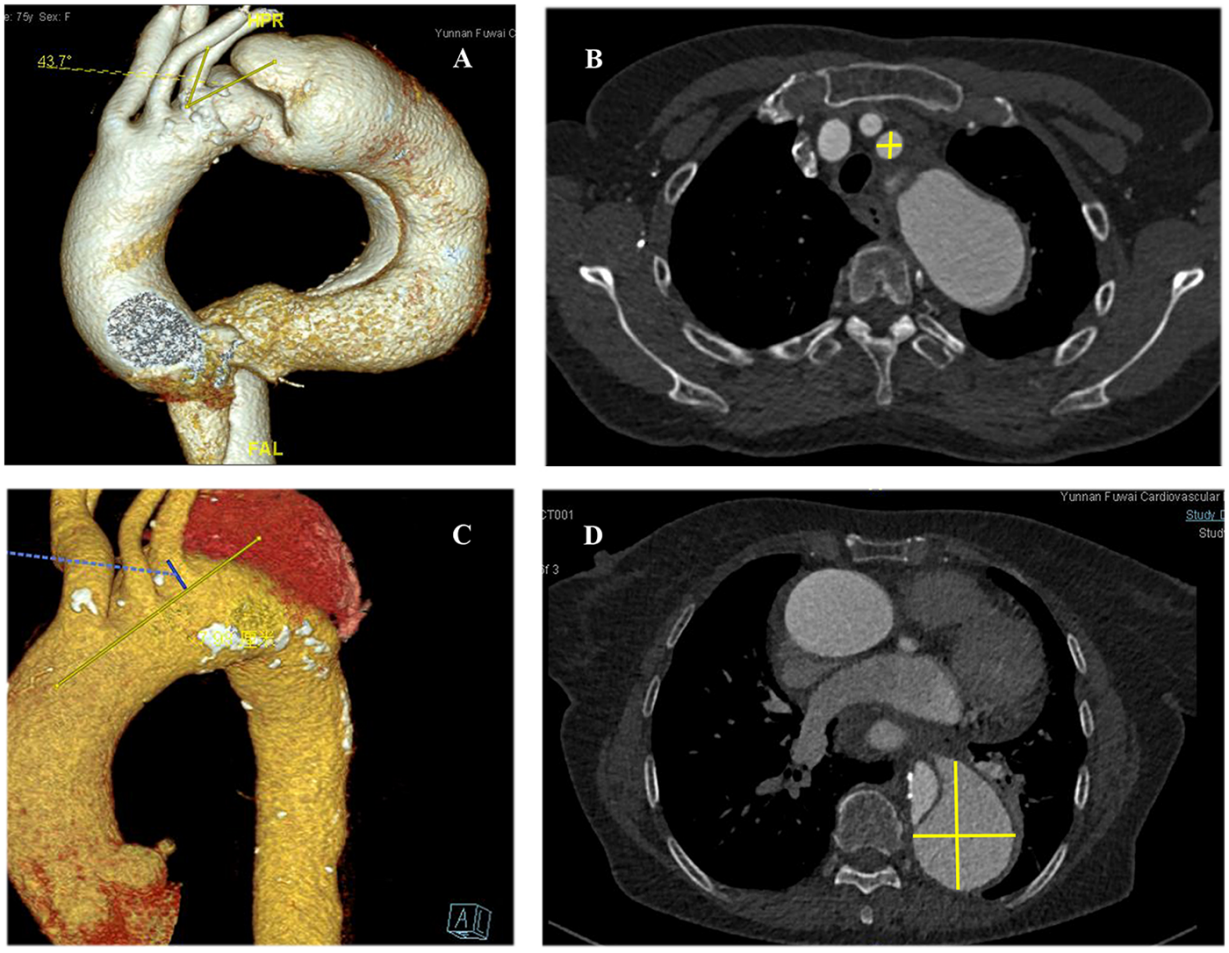
High-risk factors for type I or type II endoleaks as shown by computed tomography angiography of the aorta. (**A**) Proximal opening angle of the LSA (yellow lines) on the first preoperative view. (**B**) Diameter of the LSA (yellow crossed lines). (**C**) Distance (blue line) between the LSA and the aortic arch in the coronal section (yellow line). (**D**) Maximal diameter of the aorta (yellow crossed lines).

We measured and analyzed the proximal opening angle of the LSA and its diameter, the distance between the LSA and the aortic arch in the coronal section, and the aortic aneurysms in T1EL/T2EL patients. Univariate and multivariate logistic regression analyses showed that the proximal opening angle of the LSA (OR 3.141, 95% CI: 2.615–3.773; *P* < 0.001) was significantly associated with the incidence of T1ELs, as observed in CTA images (Figure 2A and Table 4).

**Table 4:** Multivariate risk factors for T1EL.

Factor	OR (95% CI)	*P*-value
Proximal opening angle of the LSA	3.141 (2.615–3.773)	0.001
Diameter of the LSA (mm)	0.451 (0.099–2.06)	0.432
Distance between the LSA and the aortic arch in the coronal section	0.109 (0.013–0.921)	0.001
Aneurysm size >55 mm	0.14 (0.017–1.183)	0.053

Univariate and multivariate logistic regression analyses indicated that the proximal opening angle of the LSA (OR 1.168; 95% CI: 0.222–6.152; *P* < 0.001) and the diameter of the LSA (OR 2.663; 95% CI: 2.266–3.13; *P* < 0.001) were significantly associated with the occurrence of T2ELs (Figures 2A,B, Table 5).

**Table 5:** Multivariate risk factors for T2EL.

Factor	OR (95% CI)	*P*-value
Proximal opening angle of the LSA	1.168 (0.222–6.152)	0.001
Diameter of the LSA (mm)	2.663 (2.266–3.13)	0.001
Distance between the LSA and the aortic arch in the coronal section	0.262 (0.05–1.378)	0.001
Aneurysm size >55 mm	2.426 (1.908–2.507)	0.005

## 4 Discussion

The pathologies of the thoracic aorta and its arch branches are intricate and often lead to severe conditions that pose significant challenges in diagnosis and treatment. The therapeutic strategies for these conditions remain controversial and are associated with high morbidity and mortality rates (18–20). Since the first endovascular repair of a thoracic aortic aneurysm was reported 26 years ago, endovascular approaches have largely supplanted open surgical techniques. By 2010, these methods became the gold standard for managing descending thoracic aortic aneurysms (21, 22). Due to their minimally invasive nature and improved safety profiles, TEVAR and EVAR procedures are now preferred for treating complex aortic morphologies. However, significant complications, including stroke, fatal retrograde type A dissection, and type I and type II endoleaks (T1ELs and T2ELs), continue to pose risks associated with these interventions (23–25). Previous research has identified factors such as stent graft oversizing, aortic arch curvature, and a shorter length of the proximal landing zone as high-risk contributors to the development of endoleaks (26, 27). T1ELs are characterized by high pressure and necessitate immediate intervention. The primary endovascular treatment options for T1ELs include EndoAnchors, aortic cuffs, and embolization. In contrast, T2ELs are typically low-pressure and often benign, requiring treatment only if there is an increase in sac size of at least 5 mm (1).

The early results of our research were reliable, with no 30-day mortalities or in-hospital deaths. There were no cases of retrograde type A dissection or aortic rupture. However, 14 patients were diagnosed with late T1ELs/T2ELs. Of the 56 type B IMH patients who underwent aortic repair, including open surgery and TEVAR, none developed T1ELs/T2ELs over a follow-up period of 18 ± 10 months. In some studies, fewer than 10% of IMH cases resolved (28), while 16%–47% progressed to aortic dissection (29). For type B hematoma, in-hospital mortality was reported at 4% following medical treatment and 20% following surgery (30). It has been observed that type B IMH has demonstrated a lower rate of reoperation and lower in-hospital mortality compared with TAD and TAA.

In the current study, the potential of six different parametric factors (history of smoking, aortic pathology, aneurysm size >55 mm, the proximal opening angle of the LSA, the diameter of the LSA, and the distance between the LSA and the aortic arch in the coronal section) were analyzed to distinguish high-risk T1ELs/T2ELs from thoracic aortic diseases. The importance of LSA revascularization after zone 2 TEVAR has been increasingly emphasized by recent reviews and 2022 guidelines (1, 31, 32). The highlighted evidence supports LSA revascularization as a strategy to lower the perioperative stroke rate and spinal cord ischemia (SCI). However, no direct evidence shows that LSA revascularization can reduce the occurrence of T1ELs/T2ELs. The present study attempts to retrospectively examine the LSA pathology factors in postoperative patients because the LSA coverage complications, such as left arm paralysis, stroke, or endoleaks after TEVAR/open surgery of thoracic aortic diseases, remained a big concern for surgeons. In seven of our patients, direct LSA coverage by stent grafts led to LSA regurgitation and endoleak, a condition that can result in a fatal aortic rupture. We measured the characteristics and diameter of the LSA and aorta and analyzed the data, finding that the risk factors involved the proximal opening angle of the LSA (*P* = 0.001) in T1ELs and the proximal opening angle of the LSA (*P* = 0.001), the diameter of the LSA (*P* = 0.001), and aneurysmal size >55 mm (*P* = 0.005) in T2ELs. These observations suggest that the pathological anatomy of the LSA is closely related to the occurrence of endoleaks, especially T1ELs and T2ELs. Furthermore, LSA revascularization may reduce the development of T2ELs following TEVAR. We assumed that these risk factors are associated with the hemodynamic changes resulting from the curvature of the aortic arch and the configuration of its three branches.

A significant limitation of this study is the insufficient number of prospective studies available. This lack makes it challenging to compare potential risk factors for T1ELs/T2ELs following open surgery or TEVAR. Specifically, there is a need for more data on how these risk factors relate to pathologies of the thoracic aorta and arch branches, particularly in cases with or without LSA coverage and with or without prophylactic revascularization of the LSA. Currently, the available evidence is limited to Level IV, which restricts the ability to draw robust conclusions.

## 5 Conclusions

The aim of this study was to investigate the potential of various factors (e.g., CT perfusion parameters and the anatomic structure of the aorta and its branches) in distinguishing and preventing the development of high-risk T1ELs and T2ELs after TEVAR/open surgery. Our data indicate that there was no significant difference in median survival and death rates between patients with T1EL and T2EL, meaning that these complications deserve equal attention. However, the proximal opening angle of the LSA, the diameter of the LSA, and the size of the false lumen (>55 mm) could be suggested as the highly risk factors for T1ELs and T2ELs.

Hence, appropriate stent grafts and the pathologic characteristics of the LSA should be carefully considered when a patient is undergoing an initial interventional procedure to prevent the development of T1ELs and T2ELs. To enhance patient outcomes, it is crucial to implement appropriate preoperative measures, including thorough imaging studies, such as high-resolution CT angiography, to evaluate the anatomical characteristics of the aorta and its branches. Understanding an individual patient's vascular anatomy can guide the selection of suitable stent grafts tailored to the specific dimensions and morphology of the aorta. In addition, a multidisciplinary approach involving vascular surgeons, radiologists, and interventional cardiologists can facilitate comprehensive preoperative planning. For instance, if the LSA has an excessively acute proximal opening angle or a very small diameter, alternative surgical strategies or stent graft designs may be considered to minimize the risk of endoleaks.

Despite these insights, the evidence supporting these recommendations remains limited by the paucity of randomized trials. Future research should focus on conducting well-designed studies to validate these findings and further explore the effectiveness of various preoperative strategies in preventing T1ELs and T2ELs. By addressing these issues, we can improve the safety and efficacy of TEVAR and open surgical interventions, ultimately enhancing patient outcomes.

## Data Availability

The raw data supporting the conclusions of this article will be made available by the authors without undue reservation.
